# Reply to: Common orthopaedic trauma may explain 31,000-year-old remains

**DOI:** 10.1038/s41586-023-05757-7

**Published:** 2023-03-15

**Authors:** Melandri Vlok, Tim Maloney, India Ella Dilkes-Hall, Adhi Agus Oktaviana, Pindi Setiawan, Andika Arief Drajat Priyatno, Marlon Ririmasse, I. Made Geria, Muslimin A. R. Effendy, Budy Istiawan, Falentinus Triwijaya Atmoko, Shinatria Adhityatama, Ian Moffat, Renaud Joannes-Boyau, Adam Brumm, Maxime Aubert

**Affiliations:** 1 Sydney Southeast Asian Centre, University of Sydney, Sydney, New South Wales Australia; 2 Griffith Centre for Social and Cultural Research, Griffith University, Gold Coast, Queensland Australia; 3 Australian Research Centre for Human Evolution, Griffith University, Nathan, Queensland Australia; 4 Research into Deer Genetics and Environment, RIDGE Group Inc, Ascot, Western Australia Australia; 5 Archaeology, School of Social Sciences, University of Western Australia, Crawley, Western Australia Australia; 6 School of Humanities, Languages and Social Science, Griffith University, Gold Coast, Queensland Australia; 7 BRIN, OR Arkeologi, Bahasa dan Sastra, Pusat Riset Arkeometri, Jakarta, Indonesia; 8 Faculty of Art and Design, Bandung Institute of Technology, Bandung, Indonesia; 9 Balai Pelestarian Cagar Budaya Kalimantan Timur, Samarinda, Indonesia; 10 Archaeology, College of Humanities, Arts and Social Sciences, Flinders University, Bedford Park, South Australia Australia; 11 Geoarchaeology and Archaeometry Research Group (GARG), Southern Cross University, Lismore, New South Wales Australia; 12 Palaeo-Research Institute, University of Johannesburg, Johannesburg, South Africa

**Keywords:** Anatomy, Social sciences

reply to Murphy et al. *Nature* 10.1038/s41586-023-05756-8 (2023)

We appreciate the accompanying technical Comment by Murphy et al.^1^—a group of practicing orthopaedic surgeons—on our original paper^2^. However, we strongly disagree with their conclusion that a reductionist approach was taken in the diagnosis of surgical amputation in a 31,000-year-old individual (TB1) from Borneo. We note that a complete systematic differential diagnosis was indeed completed (Extended Data Table 1); this process involved careful consideration of the most common and banal conditions first, such as accidental fracture, before considering the possibility of more rare and unusual circumstances. Through this iterative process, fracture was first eliminated as a possibility, followed by natural causes of amputation.

Surgical amputation was the remaining scenario left that completely described the characteristics that we observed in the bone. As is standard for palaeopathological analysis, a detailed description of the pathology was undertaken, including recording of the location and aspect of affected bone, the type of bone affected, the mechanism of injury, the degree of healing, complications to healing, force and fracture type. This detailed analysis means that certain aspects of trauma were excluded from the differential diagnosis due to the specific location of the injury. It was at this stage that physeal fractures, which Murphy et al.^1^ correctly recognize as common fractures in early adolescence, were disregarded from the differential diagnosis as the affected portion of the bone was at the site of the mid to distal lower third diaphysis and not near to the distal metaphyseal region (Fig. 1). We surmise that Murphy et al.^1^ may have mistaken the thin cortices of TB1’s tibia and fibula for that at the diaphyseal-to-metaphyseal transition in the bone that is naturally thin. However, with TB1, the cortices in these bones (and indeed also the left femur) are thin due to extreme atrophy that probably occurred over a number of years. We acknowledge that two-dimensional photographs and radiographs can misrepresent to readers injuries that, in reality, occur in three dimensions. Thus, we provide publicly available three-dimensional computed tomography files of the amputation.

**Fig. 1:**
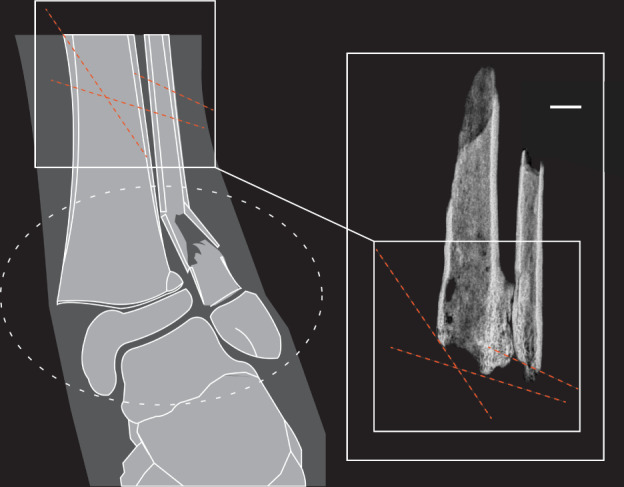
A Salter–Harris type II fracture of left tibia and fibula similar to the one presented by Murphy et al. compared with TB1’s amputation. The amputation site is more proximal (white box) to the region of Salter–Harris physeal fractures (white dashed oval). At minimum, three different angles of force are present in TB1’s amputation (red dashed lines) as opposed to one angle of force in Salter–Harris type II. Medial physis fracture of the tibia is absent in TB1 as is necessary for Salter–Harris type II classification with fibular involvement (see figure 1 of Murphy et al.^1^). Scale bar, 10.0 mm.

Moreover, the age of 6 to 9 years after surgery is a minimum age based on the minimal timing required for the completion of bone remodelling in the major long bones and, given the size of the lower limb bones, it is probable that the injury occurred in childhood. As Murphy et al.^1^ are aware, physeal stasis can have diverse traumatic origins as well as stasis of longitudinal growth in general^3,4^. Experimental animal studies demonstrate the importance for muscular activity to initiate longitudinal growth of bones through biomechanical strain. Thus, the small size of the left limb bones can be readily attributed to the existing evidence for bone atrophy related to muscle wastage^5^.

We are uncertain what Murphy et al.^1^ are referring to in their second paragraph when relating the cervical fracture to the force applied to the amputation site. Although it is possible that the cervical vertebral fracture occurred in the same event that led to the need for amputation of the lower limb, the limitations of bone response prevent us from investigating this possibility any further. Owing to the lack of empirical evidence, we refrain from speculating on the motivation or underlying cause that led to the decision to amputate. It is of course possible that the trauma described by Murphy et al.^1^ was the ultimate mechanism of injury that led to the child’s limb being surgically amputated at the location of the distal diaphysis. We clarify that we are not saying oblique fractures of the long bone shafts do not occur from blunt force trauma but are atypical in cases from an accident (excluding modern situations including transport), particularly one where the fibula and tibia were both fractured.

Murphy et al.^1^ point out in detail the requirements for their proposed scenario to have occurred but do not see the improbability of such a condition in the context of the Pleistocene tropics of Borneo. They do suggest soft tissue-only surgery as an alternative that would have involved antisepsis and debridement, which is arguably a far more sophisticated (and therefore less parsimonious) form of care that would have required a complex understanding of the anatomical basis for infection to specifically remove the infected tissue (rather than performing an entire amputation). If the fracture was not reduced through fixation, as is the case in modern Western surgical practices, a dead foot would have probably been an extreme impediment for the rugged mountainous terrain, and far more painful than a stump. Moreover, the fractured foot would have been susceptible to repeated infection as it was carried throughout the environment.

Murphy et al.^1^ incorrectly describe the remodelled bone as osteomyelitis. To support their argument, they report an anecdote in a review on the history of osteomyelitis that is from a single memoir of an American surgeon published in 1831, whose patients received treatment in hospital^6^. Osteomyelitis in the tropics is more aggressive owing to the greater diversity of the pathogens that cause osteomyelitis, the suitability of *Staphylococcus aureus*—the most common cause of osteomyelitis—to the humidity in the tropical belt and, potentially, the reduced amount of clothes worn in tropical environments increasing the infection risk of exposed wounds^7,8^. Although the mortality rate of untreated sepsis is not documented in the tropics, antibiotic-era in-hospital mortality rates in post-amputation contexts are reported to be as high as 10% and, in the Vietnam War, sepsis was attributed to 12% of deaths in surgical patients, the third leading cause of mortality in that conflict^9–11^. Osteomyelitis, both pyogenic and non-pyogenic, is readily observed in archaeological bone. In pyogenic forms, death of bone leading to sequestrum is readily observable surrounded by a shell of bone known as involucrum. Cloacae—pus draining holes—form to drain the pus from the medullary canal. Although there are circular holes in the bone, these are clearly a result of carnivore puncture and beetle scavenging marks, which are very common causes of post-mortem skeletal damage observed in Southeast Asian archaeological human skeletons (Fig. 2).

**Fig. 2:**
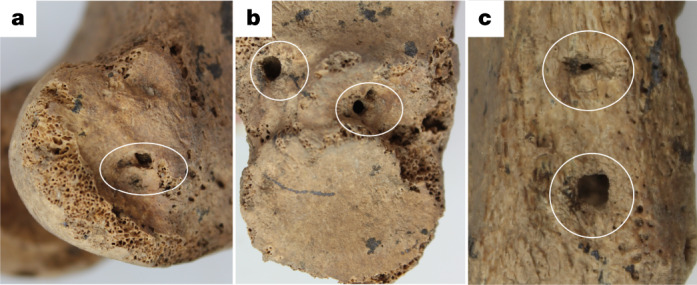
Cloacae of the right femur and tibia compared to carnivore puncture holes of the left tibia. **a**–**c**, Comparison of cloacae of the right femur (**a**) and tibia (**b**) with the carnivore puncture holes of the left tibia (**c**). The left tibia holes are clearly caused by punctures in dry bone resulting in square jagged margins to the cavities. By contrast, the margins of the holes in the right tibia and femur are rounded due to the constant remodelling process in the development of the cloacae. The femoral cloaca (**a**) also presents with a clear lytic channel consistent with infection.

The radiographs of TB1’s amputated limb (Fig. 1) show a lack of bone radiolucency associated with the development of sequestra, and the localization of radiodense bone only intermediate to the tibia and fibula is consistent with myositis ossification, and not with osteomyelitis, which will result in subperiosteal inflammation and subsequent new bone development on a more diffuse scale around the infected site. The complete lack of subperiosteal change to the tibia and fibula away from the ossified region, as well as the initiation of the subperiosteal new bone, from both the tibia and the fibula, to meet intermediately, is consequently not consistent with osteomyelitis. Moreover, chronic osteomyelitis is expected to be associated with some level of continued subperiosteal activity observed as woven bone and, in this case, the bone is entirely lamellar. Evidence of osteomyelitis in the right limb is available for comparison as well as dry bone examples from prehistoric Southeast Asia associated with and without fracture^12,13^.

We do concede the error that the medial malleolus of the right tibia is not placed in anatomical position in figure 3a of our original paper^2^. However, the aim of this figure is to represent the general completeness of the skeleton, and the relationship of the size of the left and right limbs, which we believe the figure succeeds in presenting. Given the medial malleolus is barely discernible, we believe the matter of anatomical correctness to be negligible.

## Reporting summary

Further information on experimental design is available in the Nature Portfolio Reporting Summary linked to this Article.

## Online content

Any methods, additional references, Nature Portfolio reporting summaries, source data, extended data, supplementary information, acknowledgements, peer review information; details of author contributions and competing interests; and statements of data and code availability are available at 10.1038/s41586-023-05757-7.

## Supplementary information

Reporting Summary

## Figures and Tables

**Extended Data Table 1:**
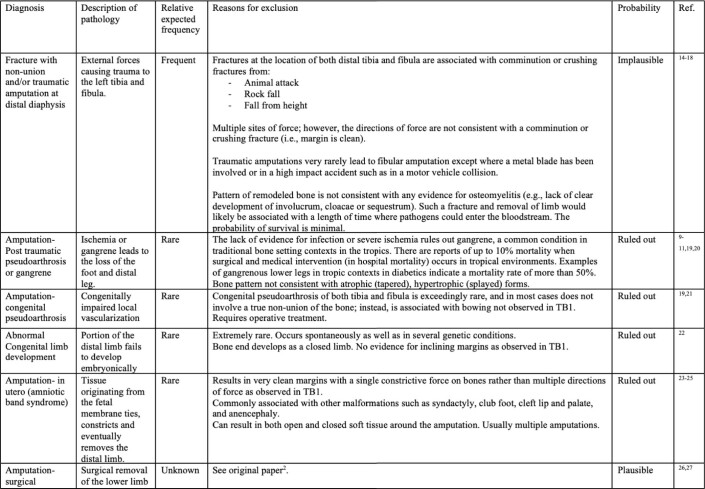
Differential Diagnosis of Tebo 1 Refs. ^14–27^

Refs. ^14–27^

## Data Availability

CT data are available at Figshare (https://figshare.com/projects/CT_Data_Tebo_TB1_Borneo_Kalimantan/150765).
